# Immune cell metabolic reprogramming in hepatocellular carcinoma: mechanisms, tumor microenvironment, and future immunotherapeutic directions

**DOI:** 10.3389/fimmu.2025.1697675

**Published:** 2026-01-12

**Authors:** Lichen Zhou, Wenjie Zhang, Zhuoran Liu, Yaming Xie, Kangyi Jiang

**Affiliations:** Department of Hepatobiliary Pancreatic Surgery, The People’s Hospital of Leshan, Leshan, China

**Keywords:** hepatocellular carcinoma, immune cell metabolism, immunotherapy, metabolic reprogramming, tumor microenvironment

## Abstract

Hepatocellular carcinoma (HCC), the most common primary liver cancer, continues to rank among the leading causes of cancer-related death despite improvements in early detection and systemic therapies. Therapeutic advances, including immune checkpoint blockade, cancer vaccines, and adoptive cell therapies, have broadened treatment possibilities. However, their efficacy and durability are often limited by immune evasion within a metabolically challenging tumor microenvironment (TME). This review consolidates current knowledge on how metabolic reprogramming in immune cells influences HCC progression, therapy resistance, and clinical outcomes. We discuss the roles of glycolysis, oxidative phosphorylation, fatty acid oxidation, and amino acid metabolism kynurenine pathways—in regulating the differentiation and function of T cells, regulatory T cells, macrophages, dendritic cells, natural killer cells, and B cells. Environmental factors such as hypoxia, lactate accumulation, adenosine signaling, and lipid remodeling act as key TME cues that suppress antigen presentation, impair cytotoxic responses, and promote immunosuppressive myeloid phenotypes. Building on these mechanisms, current strategies focus on targeting metabolic checkpoints in immune cells, reshaping the TME, and integrating metabolic modulation with checkpoint inhibitors to enhance therapeutic efficacy. In addition, candidate biomarkers (including circulating metabolites, multi-omics profiles, and liquid-biopsy indicators of immune metabolism) offer opportunities for patient stratification and dynamic monitoring. Together, these insights provide a conceptual framework in which precise modulation of immune metabolism can potentiate existing immunotherapies and guide rational combination strategies, warranting further clinical investigation to achieve sustained benefit in HCC.

## 1 Introduction

Hepatocellular carcinoma (HCC) is one of the most prevalent and lethal cancers globally, accounting for approximately 90% of all primary liver cancers (1, 2). It remains a leading cause of cancer-related mortality, with an estimated 800,000 new cases and 700,000 deaths annually (3, 4). HCC incidence is particularly high in regions with endemic hepatitis B virus (HBV) and hepatitis C virus (HCV) infections (5). It is also more frequent in individuals suffering from cirrhosis, non-alcoholic fatty liver disease (NAFLD), and alcohol-induced liver damage. Unfortunately, a substantial proportion of HCC cases are diagnosed at advanced stages, when curative interventions such as liver resection or transplantation are no longer feasible. At this stage, tumors often exhibit vascular invasion, metastasis, and an immunosuppressive tumor microenvironment (TME), collectively contributing to resistance against conventional therapies, including chemotherapy and targeted agents (6). Despite improvements in early detection methods and advancements in systemic therapies, the outlook for HCC patients remains dismal, underscoring the urgent need for the development of novel and more effective treatment options.

The challenges of treating advanced HCC stem largely from the complexity of the TME and the tumor’s capacity to evade immune surveillance (7). Conventional treatment strategies have shown limited effectiveness in altering the disease’s progression, largely because the immunosuppressive environment within the TME reduces the efficiency of systemic therapies. In recent years, there has been an increased focus on immune-based treatments, particularly immune checkpoint inhibitors (ICIs), which have demonstrated some potential in managing HCC (8–10). Nevertheless, response rates remain suboptimal, and many patients eventually develop resistance. A key factor in immune evasion in HCC is the metabolic reprogramming that occurs within immune cells in the TME (11, 12). Similar to tumor cells, immune cells undergo metabolic shifts that modulate their functional capacity. These changes in cellular metabolism often promote immune tolerance and suppression, rather than fostering an effective anti-tumor response. For instance, immune cells such as T cells and macrophages often exhibit increased glycolysis, a process that diminishes their ability to mount a robust immune response against the tumor, while simultaneously creating an environment that promotes tumor growth (13–15). These metabolic alterations are considered to be central to the immune resistance observed in HCC, highlighting the potential for targeting immune cell metabolism as a novel therapeutic strategy (16).

This review explores the role of immune cell metabolic reprogramming in HCC pathogenesis and its implications for immune evasion and therapeutic resistance. We examine critical metabolic pathways, including glycolysis, fatty acid oxidation, and amino acid metabolism, and discuss how their dysregulation fosters an immunosuppressive TME. Furthermore, we will highlight the potential of targeting immune cell metabolism as a novel approach to enhance current immunotherapeutic strategies. By understanding the interplay between immune cell metabolism and HCC progression, this review seeks to provide a comprehensive overview of how metabolic reprogramming impacts the tumor microenvironment and offers new opportunities for developing more effective immunotherapies. Ultimately, the review aims to highlight the therapeutic potential of modulating immune metabolism in HCC and provide insights into translating these strategies into clinical practice to improve patient outcomes.

## 2 Mechanisms of immune−cell metabolic reprogramming

**Figure 1:**
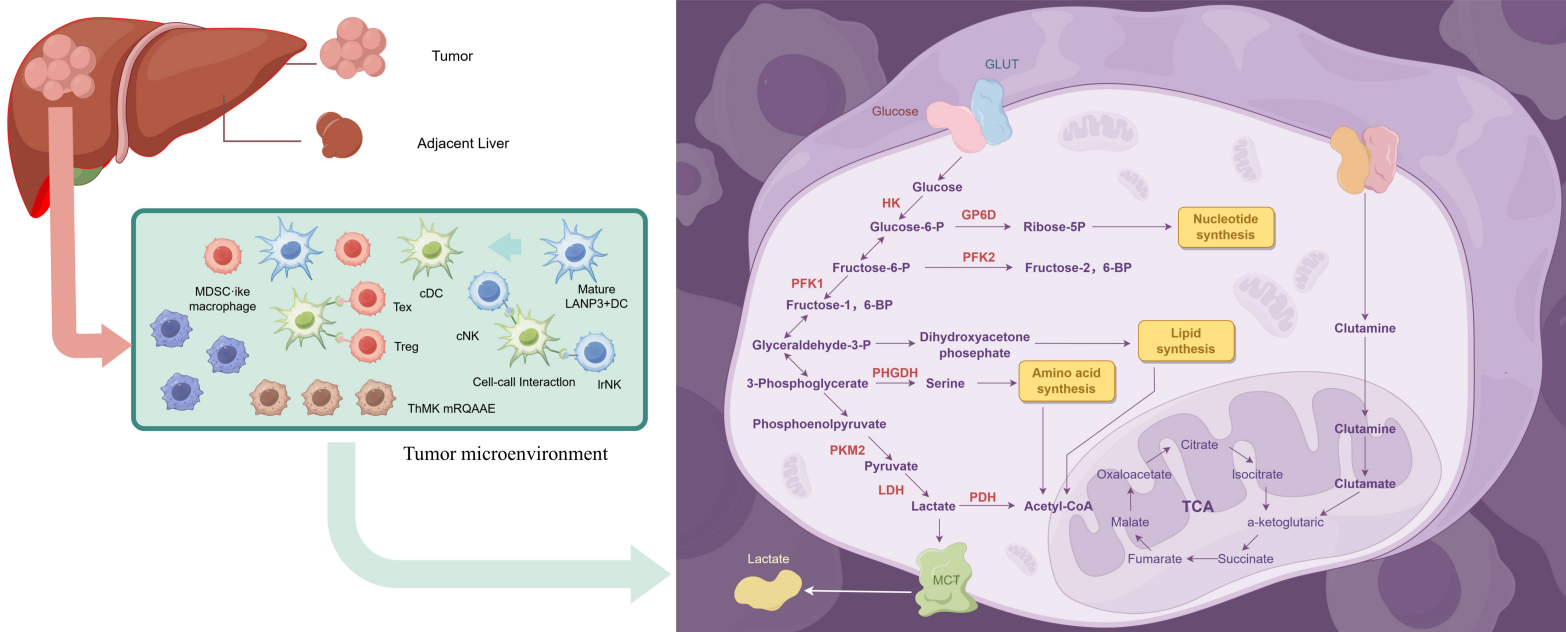
Mechanisms of immune-cell metabolic reprogramming in hepatocellular carcinoma (HCC). Schematic representation of hepatocellular carcinoma and its immune microenvironment, highlighting the metabolic reprogramming of immune cells. The figure illustrates key immune cell subsets, including myeloid-derived suppressor cells (MDSCs), cytotoxic T lymphocytes (CD8^+^ T cells), regulatory T cells (Tregs), and natural killer (NK) cells, and their interactions with tumor cells. Alterations in major metabolic pathways—such as glycolysis, oxidative phosphorylation, fatty acid metabolism, and amino acid utilization—are indicated for different immune cell types, showing how these metabolic adaptations modulate immune function and anti-tumor responses. The schematic integrates tumor development, immune suppression, and metabolic regulation to provide an overview of the mechanisms shaping immune cell function in the HCC microenvironment.

Metabolic reprogramming, in the context of both cancer and immune cells, refers to the alteration of cellular metabolism in response to the changing demands of the TME and immune challenges (17, 18). This process is typified by a shift from oxidative phosphorylation (OXPHOS) to aerobic glycolysis, known as the Warburg effect, which is a hallmark of cancer cells. The Warburg effect enables cells to maintain energy production and biosynthetic processes even in low-oxygen environments, a common feature of solid tumors (19, 20). Moreover, immune cells, like T cells, macrophages, and dendritic cells, exhibit similar metabolic reprogramming in response to activation signals, which supports their effector functions in the TME (**Figure 1**) (21–24). In cancer, this metabolic shift facilitates both tumor progression and immune evasion, highlighting the dual role of metabolic adaptation in both tumor cells and immune cells (25).

T cell activation and differentiation are tightly linked to metabolic reprogramming, particularly in glucose and fatty acid metabolism (26). Upon activation, naïve T cells rapidly shift from a resting state, dependent on oxidative phosphorylation, to a state of heightened glycolysis (27, 28). This transition is essential for the production of energy and biosynthetic precursors required for rapid proliferation and the acquisition of effector functions (29). Fatty acid oxidation (FAO) is also critical for T cell differentiation, particularly for regulatory T cells (Tregs), which depend on FAO to maintain their suppressive functions in the TME (30–32).

Numerous studies have reported that the impairment of key targets can lead to T cell metabolic reprogramming, directly influencing T cell-related immune functions. Mona et al. recruited nine patients with CARMIL2 deficiency and sixteen healthy controls, and, through comprehensive cellular metabolomics and transcriptomic profiling, observed a broad downregulation of genes associated with T cell metabolism, including pathways related to mTOR signaling, glycolysis, one-carbon metabolism, and glutamine metabolism. Subsequent *in vitro* experiments demonstrated that supplementing glutamine could partially restore T cell metabolic function (33). Based on these findings, potential therapeutic approaches targeting reduced T cell metabolic levels were proposed.

In addition, intermediates in certain metabolic pathways can also reprogram T cell immune functions. Guinevere L. et al. found that the loss of the non-classical dephytinase ABHD11 directly led to the accumulation of pentadecanoyl-lipoamide adducts, driving adaptive metabolic changes in T cells. This process impaired the activity of the 2-oxoglutarate dehydrogenase complex (OGDHc) in human CD8 lymphocytes and expanded the central memory T cell (T_++CM_) pool (34). Furthermore, Yosef et al. utilized T cell-specific ADP/ATP translocase-2 knockout mice and melanoma mouse models to confirm that the deficiency of the mitochondrial protein Ant2 in T cells bypassed typical metabolic reprogramming, inducing an activated-like metabolic state and enhancing T cell anti-tumor immunity (35). Thus, metabolic reprogramming in T cells is not only a response to activation but is intrinsic to the acquisition of specific functional capacities.

Macrophages exhibit distinct metabolic profiles depending on their polarization into either pro-inflammatory M1 or immunosuppressive M2 phenotypes (36, 37). M1 macrophages, which are induced by pathogens or pro-inflammatory cytokines such as IFN-γ, primarily rely on glycolysis to fuel their rapid activation and production of inflammatory cytokines, such as TNF-α and interleukin-6 (IL-6) (38, 39). This metabolic shift supports their pro-inflammatory functions and their role in tumor surveillance. In contrast, M2 macrophages, which are often found in the TME, favor oxidative phosphorylation and fatty acid oxidation to support tissue repair, immune suppression, and tumor-promoting functions (40). This metabolic plasticity allows macrophages to adapt to the needs of the TME, where their phenotypic state dictates their influence on tumor progression, angiogenesis, and immune suppression. Understanding the metabolic reprogramming of macrophages in the TME is critical for developing therapeutic strategies that can reprogram these cells to adopt anti-tumor phenotypes (41, 42).

As a crucial component of the TME, the metabolic relationship between macrophages and tumors is multifaceted, intricate, and reciprocal (43–45). Typically, the dominance of M2-like tumor-associated macrophages (TAM) within the TME promotes the progression of HCC, while the M1 phenotype exhibits pro-inflammatory properties and inhibits HCC progression. The polarization of TAMs toward the M1 phenotype is primarily regulated by metabolic pathways associated with aerobic glycolysis, whereas the shift of TAMs towards the M2 phenotype is characterized by oxidative phosphorylation, a process predominantly influenced by elevated lipid metabolism within the TME (46–48). Li et al. suggest that within the TME, cancer-associated fibroblasts (CAFs) influence TAMs through cytokine secretion and antigen presentation. Specifically, tumor cells activate the Syk-NF-κB signaling axis to induce the production of IL-6 and the synthesis of glutamine in fibroblasts. Subsequently, glutamine metabolism orchestrates TAM polarization, thereby promoting tumor progression and immune evasion (49). Immunosuppression within the TME is often associated with chemoresistance. One study integrated data from The Cancer Genome Atlas (TCGA) and the Molecular Signatures Database (MSigDB) to analyze lipid metabolism genes associated with macrophage infiltration in HCC. The study identified a prognostic model based on five genes (PON1, MED8, AKR1B15, MTMR2, and STARD5) that effectively reflects chemotherapeutic drug sensitivity and immune status in HCC (50). In conclusion, the relationship between TME and macrophage metabolic reprogramming in HCC is complex. Current research is focusing on the regulation of the TME metabolic profile to alter the impact of macrophages on HCC progression and immune evasion.

Dendritic cells (DCs) are pivotal in antigen presentation and the activation of T cell responses, processes that are heavily reliant on metabolic reprogramming (51). Upon activation, DCs transition from oxidative phosphorylation to glycolysis, thereby meeting the high energy demands required for antigen processing and presentation. This metabolic shift also facilitates the upregulation of co-stimulatory molecules, which are essential for effective T cell priming. Moreover, the metabolic demands of DCs during immune response initiation are influenced by the TME, where factors such as hypoxia and nutrient deprivation can profoundly alter DC function. For instance, DCs exposed to elevated lactate levels within the TME may exhibit impaired antigen presentation and a reduced capacity to activate T cells (52).

As key orchestrators of anti-tumor immunity, DCs encompass various subpopulations, including cDC1, cDC2, pDC, DC3, and tDCs. The dynamic reprogramming of these cells within the TME involves the modulation of immunosuppressive cytokines, metabolic stress, hypoxia, and lipid metabolism (53, 54). Kevin et al. reported that the expression of CD141 in the TME of melanoma patients confers a protective effect on overall survival (55). Natural killer (NK) cells, through the secretion of FLT3LG in tumors, regulate the levels of stimulatory dendritic cells (SDCs) within the tumor, thereby enhancing patient responsiveness to PD-1 blockade immunotherapy. Camille et al. elucidated the principal metabolic disturbances within tumor-infiltrating DC subpopulations. The regulation of mTOR/AMPK-dependent metabolic pathways and/or MCT1 lactate transporter activity protects DCs from the deleterious effects of tumor-derived metabolic byproducts, thus enabling robust anti-tumor immune responses (56). Therefore, targeting metabolic pathways in DCs emerges as a promising strategy to enhance the efficacy of cancer immunotherapies.

Beyond T cells, macrophages, and dendritic cells, other immune cells, such as NK cells and B cells, also undergo metabolic reprogramming in response to the TME. NK cells, which are key players in innate immunity, rely on glycolysis for rapid energy production during target cell recognition and cytotoxic activity (57). In the TME, however, NK cell function can be compromised by high levels of lactate and immunosuppressive factors, necessitating metabolic adaptations to maintain their anti-tumor activity (58). B cells, which are central to humoral immunity, also exhibit metabolic shifts upon activation, transitioning from a reliance on oxidative phosphorylation to increased glycolysis to support antibody production (59, 60). The metabolic plasticity of these immune cells in the context of HCC further highlights the complex interplay between immune cell metabolism and tumor progression, suggesting that metabolic reprogramming may represent a potential therapeutic target for enhancing anti-tumor immunity.

The key metabolic pathways involved in immune cell reprogramming include glucose metabolism (glycolysis), oxidative phosphorylation, FAO, and amino acid metabolism (61, 62). Glycolysis, which provides rapid ATP production and biosynthetic precursors, is central to the activation of many immune cells, including T cells, macrophages, and NK cells. Oxidative phosphorylation, which is more efficient in terms of energy production, is favored by memory T cells and other immune cells in less demanding environments. FAO plays a crucial role in Treg function and the polarization of macrophages and DCs. Amino acid metabolism, including glutamine metabolism, is also critical for immune cell function, particularly in the context of the TME, where nutrients such as glucose and glutamine are often limited. Additionally, cross-talk between tumor cells and immune cells in the TME, such as lactate and glutamine exchange, further modulates the metabolic reprogramming of immune cells. Understanding these metabolic pathways and their interactions within the TME provides valuable insights into potential therapeutic strategies aimed at restoring immune function and combating tumor immune evasion (**Table 1**).

**Table 1:** An overview of immune cell metabolic reprogramming and function in the tumor microenvironment of hepatocellular carcinoma.

Immune cell type	Main function in HCC	Researcher(s)	Research method	Reference number
T cells (CD8+ CTLs, CD4+ T helper cells, Tregs)	T cell activation, differentiation, and immune response in HCC. Tregs suppress immune responses in the TME.	Mona et al., Yosef et al., Guinevere L. et al.	Cellular metabolomics, In vitro experiments, Mouse models	(33–35)
Macrophages (M1, M2 phenotypes)	M1 macrophages promote inflammation and tumor surveillance, while M2 macrophages favor tumor progression and immune suppression.	Li et al., Guinevere L. et al.	In vitro experiments, Mouse models	(41, 42, 49, 50)
Dendritic cells (DCs)	Antigen presentation and T cell activation; metabolic reprogramming in response to TME conditions like lactate.	Kevin et al., Camille et al.	Metabolic pathway analysis, Tumor-infiltrating DC studies	(55, 56)
Natural Killer (NK) cells	Cytotoxic activity against tumor cells; rely on glycolysis for energy production during target cell recognition.	Lopes et al., Tian et al.	Literature review, Tumor analysis	(57, 58)
B cells	Humoral immunity; shifts from oxidative phosphorylation to glycolysis to support antibody production.	Kim et al., Seki et al.	Literature review	(59, 60)

## 3 The tumor microenvironment in hepatocellular carcinoma

HCC develops within a highly intricate TME, which is marked by a diverse array of cellular and extracellular components that interact to drive tumor growth and immune escape (63, 64). The TME in HCC is composed of malignant hepatocytes, various immune cell types, endothelial cells, fibroblasts, and the extracellular matrix (ECM). Among the immune cell populations, T cells, macrophages, dendritic cells, and Tregs play essential roles, with their activities being significantly influenced by the surrounding cellular milieu (65). Additionally, the ECM, consisting of collagen and other glycoproteins, offers structural support and affects the biomechanical characteristics of the tumor niche, impacting both cancer cell behavior and immune cell infiltration (66, 67). Endothelial cells in blood vessels and lymphatics promote tumor growth by supporting vascular formation, while fibroblasts in the tumor stroma are involved in ECM remodeling, which is a defining feature of HCC progression (68).

The liver-specific microenvironment plays a crucial role in modulating immune responses, primarily due to its ongoing exposure to harmful agents such as hepatitis viruses and alcohol (69, 70). This exposure can contribute to conditions like cirrhosis and the development of HCC. The liver houses specialized immune cells, including Kupffer cells and liver sinusoidal endothelial cells, which typically function to maintain immune tolerance toward food and microbial antigens (71, 72). However, in cases of chronic viral infections or cirrhosis, this equilibrium is disrupted. Prolonged inflammation fosters a tumor-promoting immune environment, marked by the activation of immune-suppressive pathways. This includes the recruitment of Tregs, myeloid-derived suppressor cells (MDSCs), and the release of cytokines like TGF-β and IL-10, all of which hinder effective anti-tumor immunity (73). Together, these elements contribute to the immune landscape in HCC, enabling immune evasion and the progression of aggressive tumors.

**Figure 2:**
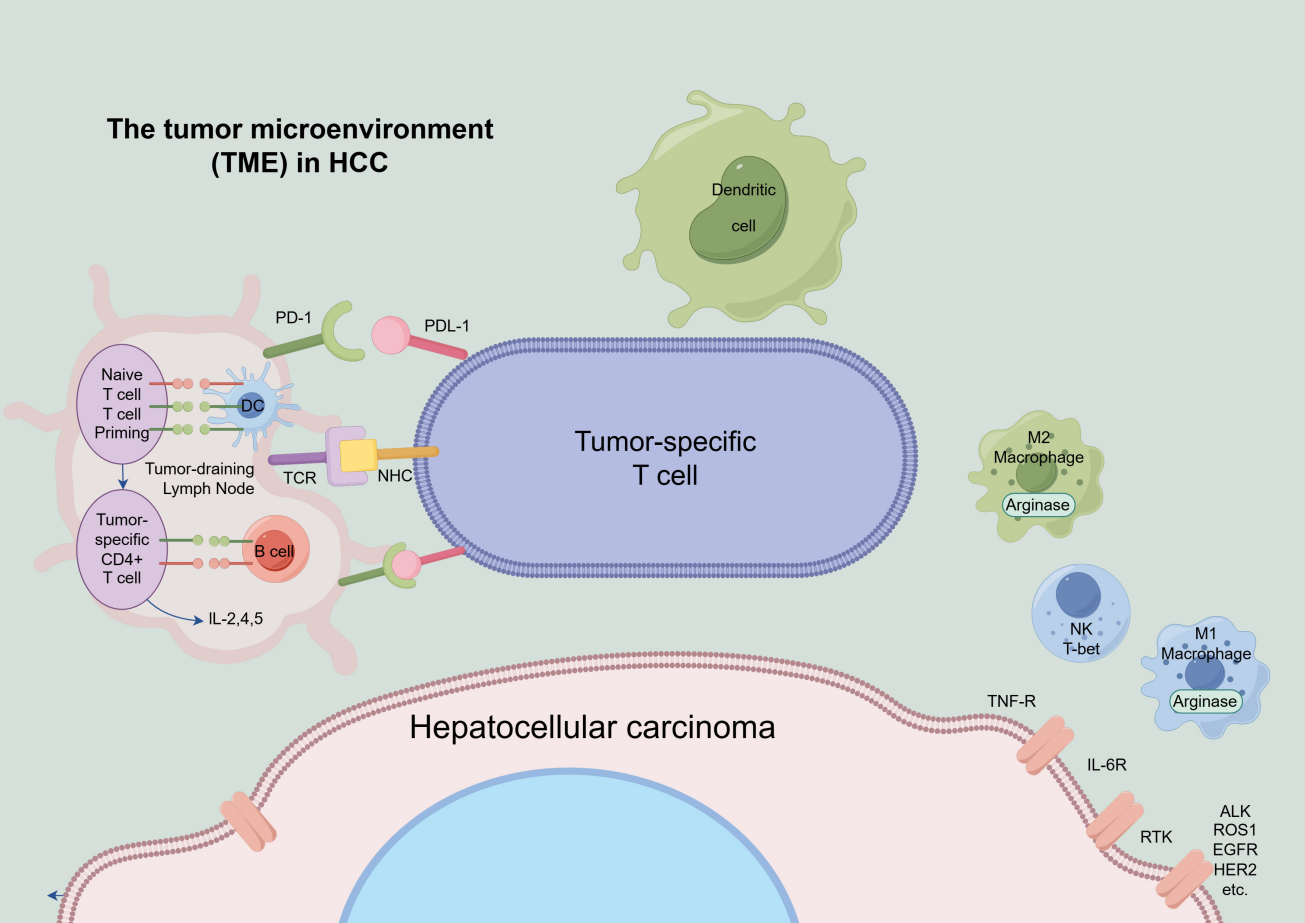
The tumor microenvironment (TME) in HCC. This schematic illustrates the major cellular and metabolic components of the HCC tumor microenvironment. Key immune cell populations—including T cells, regulatory T cells, tumor-associated macrophages, myeloid-derived suppressor cells, and NK cells—are shown alongside tumor cells and stromal elements. Immune checkpoint regulation (PD-1/PD-L1) and its impact on immune-cell dysfunction are also depicted. Together, these features outline how metabolic competition and checkpoint signaling converge to shape an immunosuppressive microenvironment that promotes HCC progression.

A key characteristic of HCC is its ability to evade immune surveillance, which plays a central role in its pathogenesis. One of the primary mechanisms through which the TME promotes immune evasion is immune cell exhaustion (74, 75). Tumor cells often take advantage of inhibitory pathways, such as the PD-1/PD-L1 and CTLA-4 interactions, which impair the function of cytotoxic T cells. The study by Hu et al. revealed elevated levels of circCCAR1 in exosomes extracted from the plasma of patients with HCC. Further molecular experiments confirmed that exosomal circCCAR1, released by HCC cells, promotes immune suppression by inducing functional exhaustion of CD8+ T cells in the HCC microenvironment (76). Moreover, the release of immunosuppressive cytokines like TGF-β and IL-10, as well as metabolites such as adenosine, further dampens the activity of effector immune cells within the TME (77, 78). The presence of Tregs and MDSCs in the tumor microenvironment also represents a key feature of immune evasion, as these cells inhibit the function of cytotoxic T cells and dendritic cells, both of which are essential for triggering effective anti-tumor responses (79). Collectively, these immune-suppressive mechanisms create a favorable environment for the progression and metastasis of HCC (**Figure 2**).

Recent breakthroughs have underscored the critical role of metabolic reprogramming in influencing immune responses within the TME of HCC. Hepatocytes and other cancer cells undergo significant metabolic shifts, such as heightened glycolysis and lactate production, even under normal oxygen conditions (80). These metabolic alterations not only fuel tumor cell growth but also profoundly affect immune cell functionality (81–83). Hypoxia, another hallmark of the TME, further exacerbates immune dysfunction by promoting the production of immunosuppressive factors, such as HIF-1α and adenosine (84, 85). Additionally, metabolic exchanges between cancer cells and immune cells, facilitated by metabolites like lactate, kynurenine, and adenosine, contribute to immune suppression (86). This metabolic dialogue creates a microenvironment conducive to tumor growth while inhibiting immune surveillance.

The TME in HCC is a dynamic and complex system that plays an essential role in tumor initiation, progression, and immune escape. The intricate interactions between tumor cells, immune cells, and the extracellular matrix support the establishment of an immunosuppressive environment. The metabolic reprogramming of both tumor and immune cells significantly contributes to the immune dysfunction seen in HCC. Building on these insights, future therapeutic strategies aimed at reprogramming the TME—through approaches like immune checkpoint inhibition, metabolic restoration, and reversal of immune cell exhaustion—hold considerable potential. Continued research into the molecular mechanisms underlying TME dynamics in HCC will be essential for developing effective immunotherapies and improving patient outcomes.

## 4 Metabolic reprogramming and the modulation of immunotherapy response in HCC

Immunotherapy has transformed the therapeutic landscape of HCC, with ICIs such as PD-1/PD-L1 and CTLA-4 blockade demonstrating significant clinical activity (87). In addition, novel approaches including cancer vaccines and adoptive cell therapies (such as CAR-T and TCR-engineered lymphocytes) are being actively investigated. Nevertheless, important hurdles persist. Both primary and acquired resistance to ICIs, the risk of immune-related toxicities, and the highly immunosuppressive TME continue to restrict long-lasting therapeutic efficacy in many patients (88). Addressing these limitations will depend on deeper mechanistic understanding of how tumor metabolism interacts with immune regulation. Effective HCC immunotherapy will require strategies that overcome metabolic- and TME-mediated resistance, optimize immune checkpoint blockade, and integrate emerging cellular and vaccine-based approaches (**Table 2**).

**Table 2:** Summary of key metabolic shifts in untreated HCC and after immune checkpoint inhibitor (ICI) therapy.

Metabolic pathway / feature	Metabolic profile in untreated HCC	Metabolic changes following ICI treatment
Glycolysis	• Tumor cells exhibit enhanced aerobic glycolysis (Warburg effect) leading to high lactate accumulation and acidification of TME. • Glycolytic competition restricts glucose availability for CD8^+^ T cells, contributing to functional exhaustion.	• Partial restoration of T-cell glycolytic activity as immune exhaustion is alleviated. • Reduced lactate-mediated immunosuppression when ICIs modulate T-cell activation.
Mitochondrial Function / Oxidative Phosphorylation (OXPHOS)	• CD8^+^ T cells show mitochondrial dysfunction, impaired biogenesis, and reduced OXPHOS. • Tregs maintain mitochondrial fitness via lipid uptake and FAO.	• ICIs may restore mitochondrial biogenesis and enhance T-cell OXPHOS. • Some patients exhibit persistent mitochondrial defects, contributing to ICI resistance.
Fatty Acid Metabolism	• Tregs and M2-like TAMs rely on FAO and lipid uptake to maintain immunosuppressive activity. • Tumor cells exploit lipid biosynthesis to support rapid growth.	• Enhanced T-cell lipid catabolism may accompany reinvigoration of effector function. • Resistance can be associated with sustained FAO in Tregs and immunosuppressive TAM populations.
Amino-Acid Metabolism (Glutamine, Arginine, Tryptophan)	• Tumor consumption of glutamine impairs effector T-cell expansion. • Arginine depletion suppresses T-cell proliferation. • IDO-mediated tryptophan catabolism generates kynurenine, promoting T-cell dysfunction.	• ICIs may partially normalize amino-acid availability by reducing immunosuppressive signals. • Elevated kynurenine levels or persistent IDO activity are linked with suboptimal responses or acquired resistance.
Hypoxia and HIF-1α Signaling	• Hypoxia intensifies immunosuppression via HIF-1α–induced VEGF, PD-L1, and adenosine production.	• Reduction in hypoxia-driven signaling may occur following effective immune activation. • Persistent hypoxia contributes to immune resistance and reduced ICI efficacy.
Lactate and TME Acidosis	• High lactate suppresses dendritic cells and CD8^+^ T cells; low pH impairs antigen presentation.	• ICIs can partially alleviate lactate-mediated suppression through T-cell reinvigoration, but tumor glycolysis often remains dominant.
Key Immunometabolic Outcomes	• Immunosuppressive TME with exhausted CD8^+^ T cells, dominant Tregs, and M2-like TAMs. • Strong metabolic competition restricts effective anti-tumor immunity.	• Reinvigoration of effector immune cells and partial remodeling of metabolic constraints. • Metabolic heterogeneity influences ICI responsiveness and resistance patterns.

Growing evidence suggests that metabolic reprogramming in immune cells is not a passive event but rather a key driver of effector activity within the TME (89, 90). For example, exhausted CD8^+^ T lymphocytes display persistent glycolytic impairment and defective mitochondrial biogenesis, resulting in diminished functionality (91). In contrast, regulatory Tregs preserve their immunosuppressive role by depending on FAO and lipid uptake. Such distinct metabolic needs constitute potential “metabolic checkpoints” that may be exploited therapeutically. Agents that enhance oxidative phosphorylation, including PGC-1α activators or drugs promoting mitochondrial biogenesis, have been shown in preclinical settings to reinvigorate CD8^+^ T-cell cytotoxicity (92). Likewise, targeting pivotal enzymes in glycolysis or FAO can alter T-cell fate decisions, thereby restraining Treg dominance in the TME. Amino acid metabolism is also emerging as a promising target: while glutamine deprivation compromises effector T-cell expansion, supplementation or metabolic redirection may restore their performance (93). Altogether, these findings highlight that reprogramming immune cell metabolism could recalibrate the immune landscape and promote effective tumor elimination.

Beyond their direct influence on immune cells, metabolic interventions designed to remodel the TME are gaining recognition as potent therapeutic adjuncts. Excess lactate generated through aerobic glycolysis in tumor cells accumulates in the extracellular space, lowering pH and impairing both dendritic cell antigen presentation and CD8^+^ T-cell cytotoxicity. Inhibiting lactate transporters or neutralizing extracellular acidity has been shown to restore effector immune activity (94, 95). Hypoxia-driven stabilization of HIF-1α enhances the production of immunosuppressive mediators such as VEGF, PD-L1, and adenosine. Interventions including oxygen supplementation, vascular normalization, or HIF-1α inhibition may therefore mitigate hypoxia-induced immune dysfunction (96). Furthermore, TAMs exhibit plasticity in response to metabolic cues—glycolytic activity favors the pro-inflammatory M1 phenotype, while lipid-fueled oxidative phosphorylation sustains the immunosuppressive M2 state (46, 47). Therapeutically, altering lipid metabolism within the TME—through reduced fatty acid uptake or by disrupting CAF-mediated glutamine synthesis—can reorient TAMs toward an anti-tumor phenotype. Taken together, manipulating the metabolic “soil” of the TME represents a strategy to simultaneously reinvigorate multiple immune cell populations and enhance anti-tumor immunity.

Given the intricate immunobiology of HCC, durable responses are unlikely to be achieved through single-agent interventions alone. A more promising therapeutic paradigm involves the integration of metabolic modulators with established immunotherapies. For example, blockade of adenosine-generating enzymes such as CD39 and CD73 has demonstrated synergy with PD-1 inhibition, thereby restoring cytotoxic T-cell function (97, 98). Suppression of indoleamine 2,3-dioxygenase (IDO)–driven tryptophan catabolism can reduce kynurenine-mediated T-cell dysfunction and augment responses to immune checkpoint blockade (99). Notably, several early-phase clinical studies are already evaluating small-molecule inhibitors of metabolic pathways in combination with ICIs, reflecting the growing translational momentum of this approach (100–102).

In parallel, the discovery of reliable biomarkers remains critical to optimizing immunometabolic therapy. Advanced multi-omics strategies, integrating metabolomics, transcriptomics, and single-cell analyses, may enable precise patient stratification and response prediction. Furthermore, non-invasive monitoring platforms such as liquid biopsy, incorporating circulating tumor DNA, exosomal RNA, and extracellular vesicle profiling, provide opportunities to dynamically track immune metabolism and therapeutic efficacy without the need for repeated tissue sampling. These tools could ultimately refine patient selection, facilitate early detection of resistance, and inform real-time adjustments in treatment strategies. Integrating multi-omics and non-invasive biomarker platforms can enhance patient stratification, monitor therapeutic responses, and guide adaptive immunometabolic treatment strategies in HCC.

## 5 Conclusion

In summary, immune cell metabolic reprogramming plays a central role in HCC pathogenesis, influencing tumor progression, immune evasion, and therapeutic resistance. Targeting metabolic pathways not only represents a novel means of reinvigorating immune surveillance but also provides a framework for overcoming the limitations of current therapies. Future HCC management may rely on rationally designed therapeutic combinations that integrate metabolic modulation with established immunotherapies, guided by predictive biomarkers. Continued mechanistic research and well-designed clinical trials will be indispensable to translate these insights into durable therapeutic benefit for patients with HCC. Looking forward, translating insights from immune cell metabolic reprogramming into clinical applications will require the development of targeted therapies that modulate specific metabolic pathways in immune cells. Rational combination strategies integrating metabolic modulators with immunotherapies, guided by predictive biomarkers, may improve patient response and overcome resistance. Continued preclinical and clinical studies will be critical to validate these approaches and bring effective metabolic-based immunotherapies into routine HCC.
